# Zinc Deficiency During Pregnancy Leads to Altered Microbiome and Elevated Inflammatory Markers in Mice

**DOI:** 10.3389/fnins.2019.01295

**Published:** 2019-11-29

**Authors:** Ann Katrin Sauer, Andreas M. Grabrucker

**Affiliations:** ^1^WG Molecular Analysis of Synaptopathies, Neurology Department, Neurocenter of Ulm University, Ulm, Germany; ^2^Institute for Anatomy and Cell Biology, Ulm University, Ulm, Germany; ^3^Department of Biological Sciences, University of Limerick, Limerick, Ireland; ^4^Health Research Institute, University of Limerick, Limerick, Ireland; ^5^Bernal Institute, University of Limerick, Limerick, Ireland

**Keywords:** Zn, microbiota, gastrointestinal, gut-brain, postpartum depression, mood disorder, immune disease, trace metal

## Abstract

Zinc is an essential trace metal for bacteria of the intestinal flora. Approximately 20% of dietary zinc – intake is used by intestinal bacteria. The microbiome has recently been described as an important factor for healthy brain function via so-called gut-brain interactions. Similarly, zinc deficiency has been associated with neurological problems such as depression, mental lethargy and cognitive impairments in humans and animal models. However, the underlying pathomechanisms are currently not well understood and a link between zinc deficiency and altered microbiota composition has not been studied. Especially during pregnancy, women may be prone to low zinc status. Thus, here, we investigate whether zinc deficiency alters gut-brain interaction in pregnant mice by triggering changes in the microbiome. To that end, pregnant mice were fed different diets being zinc-adequate, deficient in zinc, or adequate in zinc but high in zinc uptake antagonists for 8 weeks. Our results show that acute zinc-deficient pregnant mice and pregnant mice on a diet high in zinc uptake antagonists have an altered composition of gastro-intestinal (GI) microbiota. These changes were accompanied by alterations in markers for GI permeability. Within the brain, we found signs of neuroinflammation. Interestingly, microbiota composition, gut pathology, and inflammatory cytokine levels were partially rescued upon supplementation of mice with zinc amino-acid conjugates (ZnAA). We conclude that zinc deficiency may contribute to abnormal gut-brain signaling by altering gut physiology, microbiota composition and triggering an increase of inflammatory markers.

## Introduction

Zinc is one of the most prevalent trace metal ions in the body and plays a major role in the functions of the brain, immune system and endocrine system (Sauer et al., 2016). In humans, acute zinc deficiency is associated with the occurrence of skin lesions, anorexia, diarrhea, growth retardation, depressed wound healing, altered immune function, sensory impairments, and behavioral changes such as lethargy and depression (Aggett and Harries, 1979; Chasapis et al., 2012). Especially the ability to precipitate depression hints toward an interesting gut-brain interaction, given that zinc required for cognitive processes is ultimately taken up via the gastrointestinal (GI) system (Vela et al., 2015; Rafalo et al., 2016).

Low zinc status has been associated with several mood disorders including major depressive disorders and bipolar depression (Cope and Levenson, 2010). Depression is a mental disorder caused by changes in brain chemistry affecting thought processes, emotions, behaviors and overall physical health (Bondy, 2002). Interestingly, zinc status may be a biomarker of mood disorder (Styczeń et al., 2017). Several studies show that zinc deficiency induces depression while supplementing zinc improves mood as well as cognitive function in humans with depression and animal models (Piao et al., 2017).

Clinical studies have reported a reduction of blood zinc concentration in depressed patients (McLoughlin and Hodge, 1990; Maes et al., 1997; Siwek et al., 2013; Swardfager et al., 2013; Pfaender and Grabrucker, 2014) A recent meta-analysis found that depression in subjects was associated with significantly lower peripheral blood zinc concentration (Swardfager et al., 2013). Interestingly, lower zinc blood concentrations were also reported in women with postpartum depression (Etebary et al., 2010).

In animal models, acute zinc deficiency often was reported to result in depression-like behavior (Hagmeyer et al., 2014). The administration of low zinc diets leads to the development of depressive-like behavior in mice and rats (Tassabehji et al., 2008; Watanabe et al., 2010; Młyniec and Nowak, 2012; Młyniec et al., 2012, 2013a,b), assessed through measuring immobility time in the forced swim test (Corniola et al., 2008; Tassabehji et al., 2008; Whittle et al., 2009; Watanabe et al., 2010; Młyniec et al., 2012, 2013b) or tail suspension test (Młyniec and Nowak, 2012), indicating that zinc deficiency contributes to the development of this behavior. However, the underlying molecular pathomechanisms are still not well known. On a molecular level, zinc has been associated with the GPR39 receptor modulating monoaminergic and glutamatergic neurotransmission, NMDA receptor signaling (Paoletti et al., 2009), glucocorticoids (Nowak, 2001), and BDNF levels, which are all affected in depression (Młyniec et al., 2015). Further, zinc deficiency can disrupt energy metabolism and contributes to chronic inflammation (Bao et al., 2010). There is a tight interplay between zinc levels and inflammation (Prakash et al., 2015), with decreased zinc associated with a pro-inflammatory state. In particular, increased levels of circulating pro-inflammatory cytokines, which may lead to the activation of brain-resident microglia may contribute to neurobiological changes seen in depression (Wohleb et al., 2016) such as dysfunction of the monoamine system, impaired neurogenesis, and alterations in synaptic function (Horowitz and Zunszain, 2015) that result in abnormalities in regional brain activity.

While zinc deficiency negatively affects mood possibly triggering the development of depressive-like symptoms, evidence is mounting that zinc supplementation can be used to improve depressive symptoms in humans and animal models (Nowak et al., 2003; Siwek et al., 2010; Petrilli et al., 2017). Zinc supplementation exhibits antidepressant-like effects in both preclinical and clinical studies (Nowak et al., 2003; Siwek et al., 2009). In addition, the results of several randomized controlled trials show effectiveness of zinc as adjunctive therapy in depressed individuals (Nowak et al., 2003; Siwek et al., 2009; Sawada and Yokoi, 2010; Lai et al., 2012; Ranjbar et al., 2014; Solati et al., 2015). Similarly, the treatment of animal models for depression with zinc showed antidepressant effects, and zinc supplementation enhanced the effectiveness of antidepressants (Rafalo et al., 2016).

Zinc is also an essential trace metal for bacteria of the intestinal flora. A comparison between germ-free rats and rats with pathogen-free intestinal flora revealed that approx. 20% of the dietary intake of zinc was used by intestinal bacteria (Smith et al., 1972). Intriguingly, changes in gut microbiota have been implicated in a variety of conditions including depression (Dinan and Cryan, 2017). In a recent study, depression was reported associated with decreased gut microbiota richness and diversity (Kelly et al., 2016). A feedback loop between depressive states and dysregulation of the microbiome was suggested. An open question is, whether zinc deficiency alone may correspondingly alter the gut microbiome as demonstrated in mice in which chronic depression- and anxiety-like behaviors were induced by olfactory bulbectomy (Park et al., 2013).

In mice, the offspring of mice with zinc deficiency during pregnancy show autism-like behavior (Grabrucker et al., 2014, 2016). Intriguingly, in humans, maternal postpartum depression is associated with the presence of autistic traits in the offspring at 18 months of age (Salvanos et al., 2010). Pregnant women are at increased risk of developing zinc deficiency as the required daily intake almost doubles in this period (Grabrucker, 2016). Further, supplementation of folic acid, high levels of calcium and iron, as well as a diet high in phytates may lower the bioavailability of zinc (Sauer et al., 2017) during pregnancy.

Thus, here, we investigated whether dietary-induced acute zinc deficiency during pregnancy can elicit alterations in the gut microbiota composition in pregnant mice, which may translate into increased inflammatory signaling and ultimately induce changes in brain function in mice. We included a diet adequate in zinc but enriched in zinc uptake inhibitors (folic acid, phytic acid, high Ca/Fe) for comparison with a diet deficient in zinc in our study. We further investigated whether rescuing zinc levels using zinc amino-acid conjugates (ZnAA) ameliorates the observed alterations and thus may be a potential future prevention strategy. ZnAAs have advantages in overcoming especially low bioavailability of zinc (Sauer et al., 2017) and are currently available on the market as a mineral supplement for animals, where they are safe and effective and may be used in human studies in future.

## Materials and Methods

### Materials

PBS with Ca^2+^/Mg^2+^ was purchased from PAA. Paraformaldehyde was purchased from Merck, and D-Saccharose from Carl Roth. Unless otherwise indicated, all other chemicals were obtained from Sigma-Aldrich. Primary antibodies were purchased from the following companies: Thermo Fisher Scientific (ZO-1 polyclonal antibody, 61-7300, mouse specific; FABP2 polyclonal antibody, PA5-18700, mouse specific), Abcam (Anti-Claudin 3 polyclonal antibody, ab15102, mouse specific; Anti-GFAP monoclonal antibody [GF5], ab10062, mouse specific), Origene (monoclonal antibody to *Escherichia coli* LPS (J5 LPS)[Clone ID: 2D7/1], BM1091, besides *E. coli* J5 LPS, the antibody has also been found to react with *K. pneumoniae, S. sonnei*, and *S. typhimurium* LPS, the antibody detects LPS and LPS with modifications), Cell signaling Technologies (IL-6 (D5W4V) XP monoclonal antibody, #12912, mouse specific), Sigma Aldrich (Anti-Iba1/AIF1 monoclonal antibody, MABN92, mouse specific) and Merck Millipore (Anti-NR2B polyclonal antibody, 06-600, mouse specific). Alexa Fluor conjugated secondary antibodies were obtained from Invitrogen/Life Technologies Europe. Secondary HRP conjugated antibodies were purchased from Dako. Zinc amino acid complexes (ZnAAs) were obtained from Zinpro Corporation (Eden Prairie, MN, United States). Special diets for mice were purchased from Ssniff diets, Germany.

### Animals

8-week old C57BL/6JRj mice were purchased from Janvier Labs and housed upon arrival in the animal facility in plastic cages under standard laboratory conditions and provided with food and water available *ad libitum*. The housing room was maintained at 22°C, with lights automatically turned on/off in a 12 h rhythm (lights on at 7 am). After 2 weeks of acclimation, mice were divided into 4 groups: the control group (3 females) was fed with standard laboratory food (41 mg/kg zinc) (diet 1), the second group (3 females) was fed a zinc-deficient diet (19 mg/kg zinc) (diet 2). The third group received the standard laboratory food (41 mg/kg zinc), with increased levels of phytates (9.5 mg/kg), folic acid (1.9 mg/kg), Ca (1.13 mg/kg) and Fe (503 mg/kg) (diet 3). The fourth group was given diet 3 with 41 mg/kg ZnAA supplement (Sauer et al., 2017). Mice were given access to distilled, demineralized drinking water *ad libitum*. After 5 weeks, animals became pregnant and were maintained for 3 weeks of pregnancy on the respective diet. All animal experiments were performed in accordance with the guidelines and regulations for the welfare of experimental animals issued by the Federal Government of Germany and by the local ethics committee (Ulm University). The protocol used was approved by the Regierungspräsidium Tübingen, state of Baden-Württemberg, and the Ethics Committee of Ulm University (ID Number: 1257).

### Measurement of Trace Metal Concentrations

The Zn-concentration of solutions was measured by flame atomic absorption spectrometry (AAS) at the Department of Clinical Chemistry (ZE klinische Chemie) of the University Hospital Ulm using a PinAAcle 900T from Perkin Elmer.

### Immunohistochemistry

Frozen brain sections were cut at 14 μm thickness with a cryostat (Leica CM3050 S) and stored at −80°C until further use. Prior to fluorescent staining, sections were thawed for 20 min in a hydrated staining chamber, fixed in 4% paraformaldehyde (PFA)/4% sucrose/PBS for 20 min and washed three times in PBS for 5 min each. Then, sections were treated with 1x PBS with 0.2% Triton X-100 for 20 min at RT and 1 × PBS with 0.05% Triton X-100 for 10 min at RT. To prevent unspecific antibody binding, blocking was performed with blocking solution (BS) (10% FBS in 1x PBS) for 1 h at RT. Primary antibodies were diluted in BS and incubated overnight at 4°C in a humid chamber. Subsequently, sections were washed 1 × PBS with 0.05% Triton X-100 for 10 min and then incubated in a humidity chamber with secondary antibody (alexa488 or alexa568) in BS for 2 h at 37°C in darkness. After washing of tissue with 1 × PBS plus 0.05% Triton X-100 for 5 min each for three times, and additionally 5 min in 1× PBS, brain sections were counterstained with DAPI (4′,6-Diamidin-2-phenylindol) for 5 min at RT. After washing with aqua bidest, sections were mounted with Vecta Mount. Fluorescence images were obtained using an inverted confocal microscope (Zeiss LSM710) and an ImageXpress Micro Spinning Disc Confocal High-Content Imaging System (Molecular Devices), and analyses of signal intensities were performed with ImageJ 1.48r. For quantitative analysis, signals were thresholded and signal intensities of GFAP/IL-6 immunoreactivity in the vicinity of DAPI labeled cell nuclei measured using the selection tool to determine cytoplasmic protein levels. Exposure times and threshold values were equal for all groups. Brain sections from three animals per group were used and signal intensities from 10 cells from three sections per animal measured in the hippocampal CA1 + CA2 area.

### Protein Biochemistry

Liver tissue was immersed in Hepes Sucrose buffer (10 mM Hepes, 0.32 M Sucrose) and disrupted using a sonicator (Fisherbrand sonic dismembranator 120). To obtain homogenate from GI tissue, gut mucus was removed by gently squeezing it out of the intestine with the blunt point of tweezers, and tissue was submerged in PBS. Afterward the cleaned tissue was lysed in modified RIPA buffer (150 mM sodium chloride, 50 mM Tris–HCl, pH 7.4, 1 mM ethylenediaminetetraacetic acid, 1% Triton X-100, 1% sodium deoxycholic acid, 0.1% sodium dodecylsulfate) plus added protease inhibitor cocktail (complete EDTA-free Protease Inhibitor Cocktail tablets, Roche). Tissue samples in lysis buffer were disrupted with a sonicator (Fisherbrand sonic dismembrator 120). Afterward the obtained homogenate was incubated for 2 h at 4°C on a rotator. Besides semi-quantitative measurement of protein levels, the resulting lysate allows for the analysis of LPS in tissue. All materials used for lysate preparation were sterile and endotoxin-free.

LPS analysis was performed according to Sturm et al. (1984). The optional step of “baking” the nitrocellulose membrane after transfer was not performed due to simultaneous detection of ACTIN. Therefore, more diffuse LPS banding patterns are observed representing higher molecular weight LPS molecules. Using this protocol, the detection of LPS is limited to molecules having side chain lengths of approx. 30 repeat units and greater.

#### Western Blotting

Protein concentrations were determine by Bradford protein assay and Pierce BCA Protein assay, and equal concentrations loaded per lane. Proteins were separated by SDS-PAGE and blotted onto nitrocellulose membranes (GE Healthcare). Immunoreactivity was visualized using horseradish peroxidase (HRP)-conjugated secondary antibodies and Pierce SuperSignal ECL substrate (Thermo Fisher Scientific).

#### Western Blot Quantification

Evaluation of bands from Western blots (WBs) was performed using ImageJ. Three independent experiments were performed and blots imaged using a UVITEC Alliance Q9 Advanced system. The individual bands were selected and the integrated density was measured. All WB bands were normalized to β-Actin and the ratios averaged and tested for significance. Mean β-Actin signals were measured and compared between groups to exclude effects of the treatments on the proteins used for normalization. No significant differences in β-Actin levels were detected for any treatment group.

### Microbiome Analysis

#### DNA Extraction

DNA extraction of murine fecal samples was performed using the Mo Bio PowerFecal DNA Isolation Kit according to the manufacturer′s protocol. The resulting DNA concentration was measured on a Nanodrop 2000. Purity was assessed by calculating the measured A260/A280 ratio. DNA samples with an A260/A280 ratio between 1.7 to 2.0 were considered pure and subsequently used for microbiome profiling.

#### Pyrosequencing of 16S rDNA Region V3–V5

Primers were designed to target conserved sequences around the variable region 3–5 (V3–V5) of bacterial 16S rDNA. All bacterial taxonomic profiling via Illumina MiSeq was performed by Eurofins Genomics (Ebersberg, Germany).

#### Pyrosequencing Data Processing and Taxonomic Classification

All reads with errors were removed from the data set. Processing of remaining reads was performed using minimum entropy decomposition (MED), while splitting up the marker gene dataset into Operational Taxonomic Units (OTUs). Assignment of taxonomic information to each OTU was performed by BLAST aligning of cluster representative sequences to the NCBI sequence database. As a minimal requirement for reference sequences, only sequences with a sequence identity of 80% across at least 80% of a representative sequence were chosen. For each OTU a specific taxonomic assignment was transferred, selected from a set of best matching reference sequences. Using the QIIME software (version 1.8.0), taxonomic assignments and OTUs were processed further.

### Gene Expression Analysis (qRT-PCR)

Total RNA from murine brain tissue was isolated with the RNeasy Lipid Tissue Kit (Qiagen) according to the manufacturer’s protocol. Elution of total RNA was performed with sterile RNAse-free water. RNA concentration was measured with the Take3 plate on the Synergy H1 plate reader (Biotek). RNA purity was assessed by A260/A280 absorbance ratio. For each biological replicate the same amount of RNA was used per run. Quantitative RT-PCR was performed using the QuantiFast SYBR Green RT-PCR kit (Qiagen) and QuantiTect Primers (Qiagen) in a total volume of 10 μl. Thermal cycling and fluorescent detection were performed using the QuantStudio^TM^ 7 Flex Real-Time PCR System (Applied Biosystems), measuring the SYBR Green I reporter dye signal. Transcript levels were normalized to the housekeeping gene Hmbs. All cycle threshold values (ct) were calculated by the QuantiStudio Real-Time PCR software. All PCR reactions were run in triplicates.

#### qRT PCR Quantification

Relative quantification is based on internal reference genes to determine virtual mRNA levels of target genes. Ct values were transformed into virtual mRNA levels according to the formula: virtual mRNA level = 10 ^∗^ ((ct_(target)_ − ct_(standard__)_)/slope of standard curve).

### LAL Chromogenic Endotoxin Quantitation

Bacterial endotoxins in murine liver tissue samples were measured with the Pierce LAL (Limulus Amebocyte Lysate) Chromogenic Endotoxin Quantitation Kit (Thermo Fisher) according to the manufacturer′s protocol. All materials used for endotoxin measurement in samples were sterile and endotoxin-free (e.g., Fisherbrand DNase/RNase and pyrogen free 1.5 ml tubes, Eppendorf ep Dualfilter T.I.P.S^®^ SealMax). Liver samples were homogenized in Hepes Sucrose buffer using a sonic dismembrator (Fisherbrand) and diluted in endotoxin-free water for the assay. Absorbance levels of standards and samples were measured at 405 nm with the Synergy H1 plate reader (Biotek). With the help of a standard curve, endotoxin levels [endotoxin units/ml (EU/ml)] in liver samples were calculated.

### Statistics

Statistical analysis was performed with Sigmaplot 11.0 and GraphPad Prism 5. Data are shown as mean ± SEM. For multiple-group comparisons, analysis of variance (ANOVA) was performed. If groups showed significant differences *post hoc* tests for within-group comparisons were performed (Tukey test). For comparisons of two independent groups, student’s *t*-tests were used. Statistically significant differences are indicated in the figures by ^∗^*p* ≤ 0.05, ^∗∗^*p* ≤ 0.01, and ^∗∗∗^*p* ≤ 0.001.

## Results

### Low Levels of Zinc and Low Zinc Bioavailability Both Lead to Acute Zinc Deficiency in Mice

**FIGURE 1:**
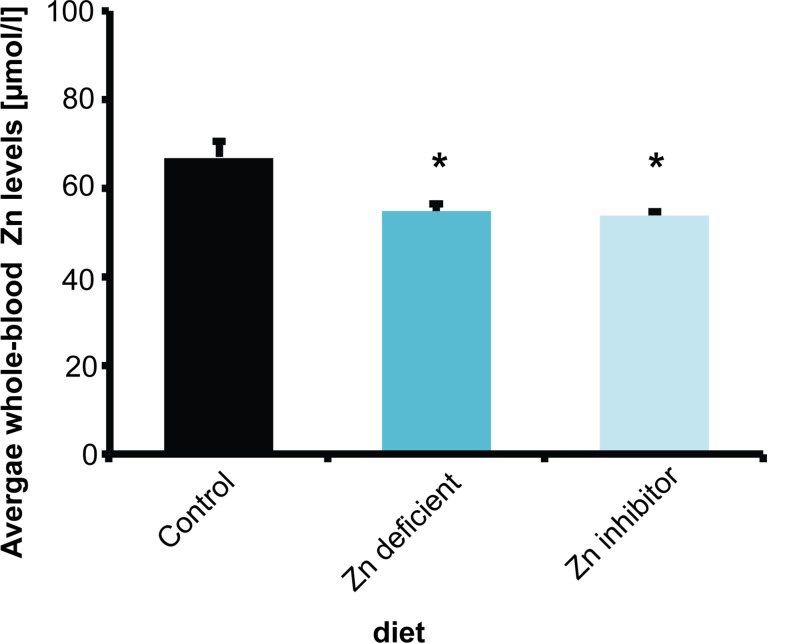
Whole-blood Zn levels of mice measured by AAS in three animals per group. Animals on a control diet (35 ppm) show average zinc levels of 66.7 μmol/l. After 8 weeks on a zinc-deficient diet (<5 ppm), significantly lower zinc levels are measured. Mice on a diet with control zinc levels (35 ppm) but the bioavailability of zinc lowered due to the presence of antagonists of absorption (Zn inhibitor) similarly to mice on a zinc-deficient diet show a significant reduction in whole-blood zinc levels.

To understand the relationship of zinc deficiency, microbiome, and brain physiology, female wild type C57BL/6 mice (10 weeks of age) were fed 3 different diets for 8 weeks. Mice received either a control diet with adequate supply of all necessary nutrients including zinc (Control diet), a diet low in zinc (Zn deficient diet) that was shown to produce mild zinc deficiency before (Grabrucker et al., 2016), or the control diet with increased levels of Zn uptake inhibitors (phytates, Ca and Fe, and folic acid) (Zn inhibitor diet). Average whole-blood zinc levels were investigated in three animals per group (Figure 1). A reduction of zinc in whole-blood does not only reflect decreased zinc levels of a fast exchanging pool of plasma zinc but indicates a zinc deficiency affecting also intracellular zinc levels of blood cells and most likely several other tissues.

The results show that animals on a standard control diet had average whole-blood zinc levels around 67 μmol/l. As expected from previous studies (Grabrucker et al., 2014, 2016), a zinc-deficient diet significantly reduced zinc levels compared to mice on the control diet (one way ANOVA, *F*_(__2_,_6__)_ = 8.739, *p* = 0.017, *Post hoc* analysis: Control vs. Zinc deficient, *p* = 0.0461). Interestingly, the presence of additional phytates, folic acid, and Ca and Fe ions (zinc uptake antagonists) also led to a significant reduction in zinc levels (Figure 1) (Control vs. Zinc inhibitor, *p* = 0.0307). Thus, the presence of high phytate levels such as found in a plant-rich diet and folic acid and mineral (Ca, Fe) supplements significantly lowers zinc bioavailability in the diet to a level comparable to a zinc depleted diet in these experiments.

### Low Dietary Levels or Bioavailability of Zinc Result in Altered Microbiota Composition in Pregnant Mice

**FIGURE 2:**
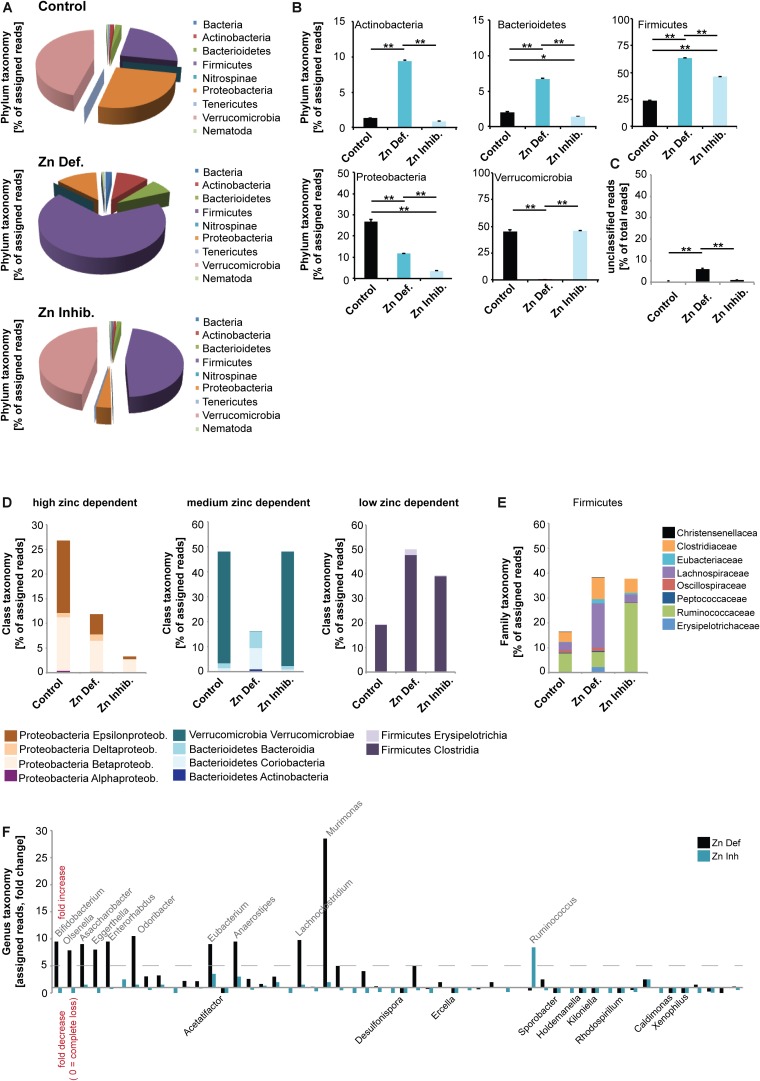
DNA was extracted from feces from three animals per group and microbiota composition analyzed using 16s microbiome profiling. **(A,B)** On phylum level, significant differences were detected. **(A)** Using the obtained sequence information, single species and their relative amounts were identified. Each phylum was assigned a color. Both, relative abundance and the composition of species are different between the three groups. Verrucomicrobia is the most prevalent phylum in control mice. On a zinc-deficient diet, Verrucomicrobia levels are reduced and Firmicutes are the most prevalent phylum. Mice on the Zn inhibitor diet show an intermediate microbiome with Verrucomicrobia levels similar to controls but also an increase in Firmicutes. **(B)** While no difference was found between the control and Zn inhibitor diet, mice on Zn deficient diet had significantly higher levels of Actinobacteria compared to controls and mice on the Zn inhibitor diet. Levels of Bacterioidetes were significantly increased in mice on Zn deficient diet compared to the control and Zn inhibitor diet. Zn deficient and Zn inhibitor diets significantly increase the level of Firmicutes. The increase was significantly higher in mice on a Zn deficient diet. The level of Proteobacteria significantly decreases in mice on Zn deficient and Zn inhibitor diet. The decrease was significantly higher in mice on a Zn inhibitor diet. Zn deficient but not Zn inhibitor diet significantly reduces the amount of Verrucomicrobia. **(C)** The number of unclassified reads was significantly higher in mice on a zinc-deficient diet. **(D)** Left panel: Proteobacteria are highly sensitive to zinc depletion with a significant decrease occurring both on Zn deficient and Zn inhibitor diet. Although Epsilonproteobacteria and Betaproteobacteria are significantly reduced by zinc restriction, Deltaproteobacteria increase. Middle panel: Verrucomicrobia are not altered under the Zn inhibitor diet, but significantly reduced in mice on a Zn deficient diet. Bacterioidetes, especially Bacteriodia, significantly increase on a Zn deficient diet. Right panel: Firmicutes significantly increase under Zn deficient and Zn uptake inhibition. **(E)** Lachnospiraceae are significantly increased on a Zn deficient diet. Ruminococcaceae are more characteristic of a Zn inhibitor diet. **(F)** A more than fivefold increase in mice on Zn deficient or Zn inhibitor is found in the genus Bifidobacterium (*Bifidobacterium pseudolongum)*, Olsenella, Asaccharobacter (*Asaccharobacter WCA-131-CoC-2*), Eggerthella (*Eggerthella YY7918*), Enterorhabdus (*Enterorhabdus mucosicola*), Odoribacter (*Odoribacter laneus*), Eubacterium *(Eubacterium plexicaudatum*), Anaerostipes (*Anaerostipes butyraticus*), and Lachnoclostridium (*Lachnoclostridium scindens*). The most significant and largest increase was seen in the genus Murimonas (*Murimonas intestine*) under Zn deficient conditions. Ruminococcus (*Ruminiclostridium cellobioparum*) display the largest increase under Zn uptake inhibition. Bacteria of the genus Acetatifactor, Desulfonispora, Ercella, Sporobacter, Holdenmanella, Kiloniella, Rhodospirillum, Caldimones, and Xenophilus were highly reduced or absent in mice on Zn deficient and Zn inhibitor diet.

Next, to investigate whether low dietary zinc availability over a period of 8 weeks is sufficient to induce alterations in the gut microbiota composition of mice, we performed pyrosequencing of 16S rDNA of fecal samples. A microbiome profile of mice from all different groups was established (Figure 2). The results show that both Zn deficient diet and the Zn inhibitor diet lead to significantly different microbiota composition in pregnant mice (Figure 2A). However, the microbiota composition was also different between Zn deficient diet and the Zn inhibitor diet. Thus, in the presence of zinc uptake antagonists, some microbiota are still successfully able to compete for zinc. In general, on phylum level, Verrucomicrobia were the most prevalent in control mice. In mice on a zinc-deficient diet, Verrucomicrobia levels are dramatically reduced and Firmicutes become by far the most prevalent microbiota phylum. In mice on a diet with adequate zinc levels but the presence of zinc uptake antagonists, Verrucomicrobia levels are similar to control mice but Firmicutes increase as well. A significantly higher number of different species was detected in the microbiome of animals on a zinc-deficient diet compared to animals on control diet (one way ANOVA, *p* = 0.002, *Post hoc* analysis: Control vs. Zinc deficient, *p* = 0.0167) (data not shown).

In detail (Figure 2B), a significant difference was found in the phylum Actinobacteria (one way ANOVA, *p* = 0.00003). While no difference was found between control and Zn inhibitor diet, mice on Zn deficient diet had significantly higher levels of Actinobacteria compared to controls and mice on Zn inhibitor diet (Tukey *post hoc* analysis: Control vs. Zinc deficient, *p* < 0.01; Control vs. Zinc inhibitor, *p* < 0.01). In addition, levels of Bacterioidetes were significantly different (one way ANOVA, *p* = 0.00004). Mice on Zn deficient diet showed significantly increased levels compared to control and Zn inhibitor diet (Tukey *post hoc* analysis: Control vs. Zinc deficient, *p* < 0.01; Zinc deficient vs. Zinc inhibitor, *p* < 0.01). Further, both Zinc deficient and Zinc inhibitor diets significantly increase the level of Firmicutes compared to the control diet (one way ANOVA, *p* = 0.00003; Tukey *post hoc* analysis: Control vs. Zinc deficient, *p* < 0.01; Control vs. Zinc inhibitor, *p* < 0.01). The increase was significantly higher in mice on a zinc-deficient diet (Zinc deficient vs. Zinc inhibitor, *p* < 0.01).

In contrast, both Zinc deficient and Zinc inhibitor diets significantly decrease the level of Proteobacteria compared to the control diet (one way ANOVA, *p* = 0.0003; Tukey *post hoc* analysis: Control vs. Zinc deficient, *p* < 0.01; Control vs. Zinc inhibitor, *p* < 0.01). The decrease was significantly higher in mice on a zinc inhibitor diet (Zinc deficient vs. Zinc inhibitor, *p* < 0.01). Zinc depleted diet, but not the diet with adequate zinc levels and presence of zinc uptake inhibitors dramatically reduced the amount of Verrucomicrobia (one way ANOVA, *p* = 0.0006; Tukey *post hoc* analysis: Control vs. Zinc deficient, *p* < 0.01; Zinc deficient vs. Zinc inhibitor, *p* < 0.01). The number of unclassified reads was significantly higher in mice on a zinc-deficient diet (Figure 2C).

Based on the alterations observed on the phylum level, we conclude that the phylum Proteobacteria is very sensitive to zinc depletion as already lower bioavailability of zinc leads to a significant decrease of this phylum. However, within the phylum, not all classes respond similarly. For example, while Epsilonproteobacteria and Betaproteobacteria are highly reduced by zinc restriction, Deltaproteobacteria slightly increase (Figure 2D). In contrast, the phylum Firmicutes thrives under low zinc conditions (Figure 2D). Verrucomicrobia can cope with low bioavailability of zinc and seem to have mechanisms of zinc intake that successfully compete with the presence of uptake inhibitors. However, under zinc-depleted conditions, the presence of the phylum in the gut microbiome is drastically reduced. Bacterioidetes, especially the class Bacteriodia, are more successful in populating the gut microbiome under zinc depletion (Figure 2D).

Given that the loss of Proteobacteria and an increase in Firmicutes occurs in both zinc restricted diets, we closer analyzed the composition of Firmicutes to investigate, which bacteria increase in numbers or are newly found in the GI tract of these mice (Figure 2E). We found that especially the Firmicutes family Lachnospiraceae benefits from zinc depleted conditions, while Ruminococcaceae are more characteristic for a diet high in zinc uptake antagonists. Changes in the occurrence of members of the Lachnospiraceae have been associated with chronic inflammation of the gut (Manichanh et al., 2006; Berry et al., 2012).

Finally, we analyzed the different microbiomes on genus level (Figure 2F). Among 223 different genera, we highlight those that show a more than 5-fold increase in mice on zinc-deficient or zinc inhibitor diet and those being lost from the microbiome in both zinc deficient or zinc inhibitor diets. We found an increase in the genus Bifidobacterium mostly due to an increase in *Bifidobacterium pseudolongum*, Olsenella, Asaccharobacter due to an increase in *Asaccharobacter WCA-131-CoC-2*, Eggerthella due to an increase in *Eggerthella YY7918*, Enterorhabdus due to an increase in *Enterorhabdus mucosicola*, Odoribacter due to an increase *Odoribacter laneus*, Eubacterium due to an increase in *Eubacterium plexicaudatum*, Anaerostipes due to an increase *Anaerostipes butyraticus*, and Lachnoclostridium due to an increase *Lachnoclostridium scindens*. A very large increase was found in the genus Murimonas, especially *Murimonas intestine* under zinc-deficient conditions, which may be a potent biomarker for zinc deficiency (Figure 2F). The genus Ruminococcus, especially *Ruminiclostridium cellobioparum* shows a specific increase in mice on the zinc inhibitor diet (Figure 2F).

The genus Turicibacter, Allobaculum, Marvinbryantia, and Butyrivibrio only appeared in the microbiome of mice on zinc-deficient or zinc inhibitor diets (not shown).

### Low Dietary Levels or Bioavailability of Zinc Result in Altered Gut Physiology

*Enterorhabdus mucosicola* was originally isolated from inflamed ileal samples of TNF^deltaARE^ mice (Clavel et al., 2009). In addition, both *E. plexicaudatum* and *E. mucosicola* are associated with inflamed gut mucosa and intestinal epithelial barrier dysfunction. Further, *Egerthella YY7918* is closely related to the type strain *Eggerthella lenta VPI0255*. *Eggerthella lenta* has been to be part of the normal human intestinal microbiome and has been associated with infections of the gastrointestinal tract (Gardiner et al., 2015). Therefore, next, we evaluated markers of intestinal physiology and “leakiness”.

**FIGURE 3:**
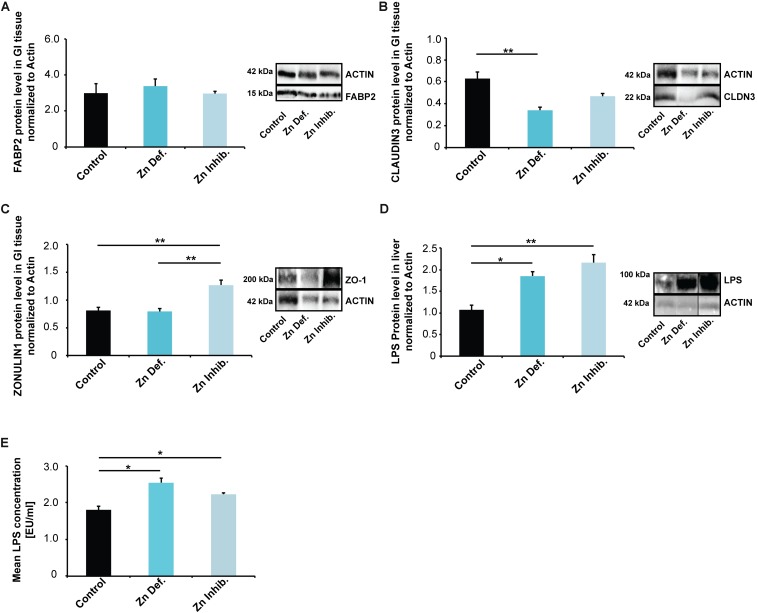
**(A–C)** Gastro-intestinal epithelium was isolated from three mice per group and protein lysates analyzed using Western blotting. **(A)** No significant differences in FABP2 were detected. **(B)** Mice on a Zn deficient diet had significantly reduced levels of CLAUDIN3. **(C)** ZONULIN1 was significantly increased in mice on Zn inhibitor diet. **(D)** Liver tissue was isolated from three mice per group and protein lysates analyzed for the levels of *E. coli* lipopolysaccharide (LPS) using Western blotting. A significant increase in liver LPS is visible in mice on a Zn deficient and Zn inhibitor diet. **(E)** LPS levels were measured by LAL assay from three mice per group in triplicates. The results show significantly increased LPS levels in liver lysate from mice on Zn deficient and Zn inhibitor diet compared to controls.

We selected three markers, FABP2 (Intestinal fatty acid-binding protein 2), CLAUDIN3, and ZONULIN1 (HP1) (Figures 3A–C). FABP2 is a cytosolic protein found in small intestine epithelial cells where it participates in the uptake, intracellular metabolism, and transport of long-chain fatty acids. CLAUDIN3 is a cell adhesion protein found at tight junctions between gut epithelial cells. ZONULIN1 is a physiological modulator of intercellular tight junctions and alterations in the ZONULIN regulated pathways have been associated with both intestinal and extra-intestinal autoimmune and inflammatory disorders (Fasano, 2011). ZONULIN1 and FABP2 have been proposed as markers of gut dysbiosis and gut permeability integrity (Stevens et al., 2018), with a decrease in FABP2 and an increase in ZONULIN1 linked to increased gut permeability (Fasano, 2012).

While we found no significant differences in the lysate from the entire small intestine for FABP2 (Figure 3A), mice on a zinc-deficient diet had significantly reduced levels of CLAUDIN3 (Figure 3B) (one way ANOVA, *p* = 0.0084; Tukey *post hoc* analysis: Control vs. Zinc deficient, *p* = 0.0068), and mice on Zinc inhibitor diet showed a trend toward a reduction. ZONULIN1 was significantly increased in mice on Zinc inhibitor diet (Figure 3C) (one way ANOVA, *p* = 0.0046; Tukey *post hoc* analysis: Control vs. Zinc inhibitor, *p* = 0.0082) compared to controls and mice on zinc-deficient diet (Tukey *post hoc* analysis: Zinc deficient vs. Zinc inhibitor, *p* = 0.0069).

The detoxification of microbial products from gut-derived microbiota is a function of the liver. Analyzing liver tissue of mice for the levels of *E. coli* lipopolysaccharide (LPS), we found a significant increase in liver LPS in mice on a zinc-deficient and zinc inhibitor diet (one way ANOVA, *p* = 0.0054; Tukey *post hoc* analysis: Control vs. Zinc deficient, *p* < 0.05; Control vs. Zinc inhibitor, *p* < 0.01) (Supplementary Figure S1A and Figure 3D). To validate this data, we additionally measured *E. coli* LPS (endotoxin) levels in mouse liver using a Limulus Amebocyte Lysate (LAL) based assay. The results confirm significantly increased levels of LPS in liver of mice on a zinc-deficient and zinc inhibitor diet (one way ANOVA, *p* = 0.047; *post hoc* analysis: Control vs. Zinc deficient, *p* = 0.041; Control vs. Zinc inhibitor, *p* = 0.0143) (Supplementary Figure S1B and Figure 3E). Thus, altered microbiota composition together with an increased intestinal permeability may be responsible for increased translocation of bacterial LPS into the systemic circulation (Szabo et al., 2010).

### Low Dietary Levels or Bioavailability of Zinc Result in Increased Inflammation in Pregnant Mice

**FIGURE 4:**
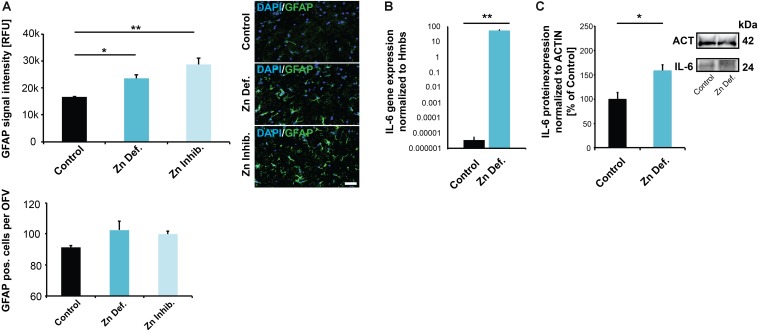
**(A)** Brain sections from three animals per group were used for immunohistochemistry. DAPI (labeling cell nuclei) and GFAP was visualized and fluorescent signal intensities from 10 cells in the hippocampus from three sections per animal measured. Mean values show the average of three animals per group. Upper panel: A significant increase in GFAP expression levels can be detected in the brains of mice subjected to zinc deficiency and lowered bioavailability (Zn Inhibitor) compared to control mice. Lower panel: No significant difference in the number of GFAP positive cells per optic field of view (OFV) was found. Scale bar = 100 μm. **(B)** Whole-brain total RNA lysate from three animals per group was used to analyze the expression of IL-6 on gene level normalized to Hmbs. A significantly higher IL-6 expression was found in the brain of zinc-deficient mice. **(C)** Whole-brain protein lysate from three animals per group was used to analyze IL-6 expression on protein level normalized to ACTIN. Significantly higher IL-6 levels are found in the brain of zinc-deficient mice.

Given that an increase of ZONULIN1 and loss of CLAUDIN3 have been associated with increased gut permeability and inflammation, we next analyzed whether we can detect signs of (neuro)inflammation in the brain of pregnant mice with low zinc status. To that end, we analyzed tissue for the expression levels of GFAP and IL-6. Glial fibrillary acidic protein (GFAP) is an established marker for the activation of astrocytes following injury or stress (Zhang et al., 2017). Expression of IL-6 was reported being induced in both astrocytes and microglia in response to LPS and increased levels in the brain are related to inflammatory and pathological situations (Erta et al., 2012). Our results show a significant increase in GFAP expression levels in brains of mice subjected to zinc deficiency and lowered bioavailability in the diet compared to control mice (one way ANOVA, *p* = 0.0044; Tukey *post hoc* analysis: Control vs. Zinc deficient, *p* = 0.0447; Control vs. Zinc inhibitor, *p* = 0.0036) (Figure 4A). The number of GFAP positive cells was not significantly altered (Figure 4A, lower panel).

The levels of IL-6 were significantly elevated in brain tissue of both mice on zinc-deficient diet and mice with sufficient dietary zinc levels but presence of zinc uptake inhibitors compared to controls (one way ANOVA, *p* = 0.028; Tukey *post hoc* analysis: Control vs. Zinc deficient, *p* = 0.04547; Control vs. Zinc inhibitor, *p* = 0.038557) (Supplementary Figure S2A). However, measuring IL-6 levels in brain sections by IHC suffers from specificity and sensitivity issues. Therefore, to further validate the data from IHC, we assessed IL-6 expression on the transcription level. The results show a significantly higher concentration of IL-6 mRNA in the brain of pregnant mice on a zinc-deficient diet (*t*-test, *p* = 0.0041) compared to controls (Figure 4B). Besides, we detected the increased expression of further inflammatory marker genes in pregnant mice on a zinc-deficient diet such as significantly higher levels of IL-1b (*p* = 0.0034), S100β (*p* = 0.0185), and CCL2 (*p* = 0.0109, *t*-tests) (Supplementary Figure S2B). The increased IL-6 transcription translates into increased IL-6 on protein level, further quantified by western blotting (*t*-test, *p* = 0.0283) (Figure 4C).

Although more extensive analyses need to be done in the future, the obtained data hint at a physiologic relevant impact of dietary zinc restriction during pregnancy on the brain of pregnant mice.

### Supplementation of Maternal Diet With ZnAAs Prevents Several Alterations Induced by Low Bioavailability of Zinc

Recently, we have investigated the mechanisms of uptake and absorption of ZnAAs (Sauer et al., 2017). ZnAAs are zinc supplements with zinc stably conjugated in an amino acid backbone. These ZnAAs were taken up by cells, not through classical zinc transporter proteins but amino acid transporters. Therefore, ZnAAs showed a significant advantage compared to inorganic zinc salts such as ZnCl_2_ as a supplement, since ZnAAs do not compete with other metals for zinc transporters and seem to be less accessible for folic acid or phytic acid. Therefore, here, we supplemented a diet rich in zinc uptake antagonists (diet 3, Zn Inhibitor) using ZnAAs to investigate whether the observed changes can be prevented by dietary zinc supplementation.

**FIGURE 5:**
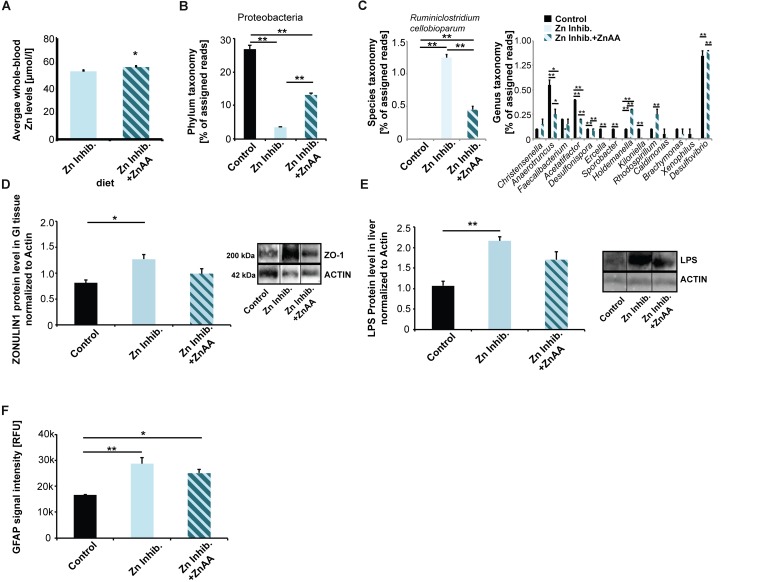
**(A)** Whole-blood Zn levels of mice measured by AAS in three animals per group. Mice were fed ZnAA supplemented diet (diet 4, Zn Inhibitor + ZnAA). At the end of pregnancy (8 weeks treatment in total), mice on a diet with control zinc levels (35 ppm) but bioavailability of zinc lowered due to the presence of antagonists of absorption (Zn inhibitor) show significantly lower whole-blood zinc levels compared to mice on the same diet with the addition of ZnAAs. **(B)** Pyrosequencing of 16S rDNA of fecal samples shows that the abundance of Proteobacteria is significantly higher in mice on Zn inhibitor + ZnAA diet compared to mice on Zn inhibitor diet. The levels were still significantly lower compared to mice on the control diet. **(C)** Left panel: On species level, the almost 10-fold increase in *Ruminiclostridium cellobioparum* seen in mice on Zn Inhibitor diet was partially prevented. Right panel: Bacteria of the genus Christensiella, Anaerotruncus, Faecalibacterium, Acetatifactor, Desulfonispora, Ercella, Sporobacter, Holdenmanella, Kiloniella, Rhodospirillum, Caldimones, Brachymonas, Xenophilus, and Desulfovibrio were highly reduced or absent in mice on Zn inhibitor diet. Supplementation with ZnAA was able to prevent the reduction/loss for Christensiella, Anaerotruncus, Faecalibacterium, Acetatifactor, Desulfonispora, Holdenmanella, Rhodospirillum, Brachymonas, and Desulfovibrio. **(D)** GI epithelium was isolated from three mice per group and protein lysates analyzed using Western blotting. Supplementation with ZnAA was able to prevent the significant increase of ZONULIN1 observed in mice on Zn Inhibitor diet. Data for Control and Zn Inhibitor diet has been reused from Figure 3C. **(E)** Liver LPS is significantly increased in mice on Zn inhibitor diet, but not in mice on Zn inhibitor + ZnAA diet. **(F)** Brain sections from three animals per group were used for immunohistochemistry. DAPI (labeling cell nuclei) and GFAP or IL-6 were visualized and fluorescent signal intensities from 10 cells in the hippocampus from three sections per animal measured. Mean values show the average of three animals per group. **(F)** A significant increase in GFAP expression levels can be detected in the brains of mice on the Zn Inhibitor diet compared to control mice. Dietary supplementation with ZnAA slightly, but non-significantly decreased GFAP expression levels.

Mice were fed a ZnAA supplemented diet (diet 4, Zn Inhibitor + ZnAA) for 8 weeks. At the end of pregnancy, the average whole-blood zinc levels were investigated and compared to mice on the same diet with low bioavailability of zinc but without zinc supplementation and controls. The results show that the addition of ZnAAs to the diet leads to significantly higher zinc levels compared to mice on the diet with low bioavailability of zinc (*t*-test, *p* = 0.0225) (Figure 5A) and no significant difference can be seen compared to controls (*p* = 0.0736).

Next, we examined if alterations in microbiota composition are normalized by zinc supplementation. We again performed pyrosequencing of 16S rDNA of fecal samples and investigated the microbiome of mice fed ZnAA supplemented diet (Figure 5B). The results show that on phylum level, the abundance of Proteobacteria was significantly higher in mice supplemented with ZnAA compared to mice on a diet with lowered bioavailability of zinc. However, the levels did not reach those of mice on a control diet (one way ANOVA, *p* = 0.0004; Tukey *post hoc* analysis: Control vs. Zinc Inhibitor, *p* < 0.01; Control vs. Zinc inhibitor + ZnAA, *p* < 0.01; Zinc Inhibitor vs. Zinc inhibitor + ZnAA, *p* < 0.01). We could not detect further rescue effects on phylum level. Instead, supplementation with ZnAA seemed to generate a new microbiota composition, which was significantly different from controls and mice on the Zn Inhibitor diet (Supplementary Figure S3A). Therefore, we focused on the most prominent alterations observed in mice on a diet with low bioavailability of zinc on genus and species level and compared these to mice on the same diet, but with supplementation of ZnAAs. An almost 10-fold increase in Ruminococcus (*R. cellobioparum*) was observed under the low bioavailability of zinc. Supplementation with ZnAAs was able to partially prevent this increase (Figure 5C, left panel) (one way ANOVA, *p* = 0.0005; Tukey *post hoc* analysis: Control vs. Zinc Inhibitor, *p* < 0.01; Control vs. Zinc inhibitor + ZnAA, *p* < 0.01; Zinc Inhibitor vs. Zinc inhibitor + ZnAA, *p* < 0.01). Bacteria of the genus Christensiella, Anaerotruncus, Faecalibacterium, Acetatifactor, Desulfonispora, Ercella, Sporobacter, Holdenmanella, Kiloniella, Rhodospirillum, Caldimones, Brachymonas, Xenophilus, and Desulfovibrio were highly reduced or absent in mice on Zn inhibitor diet. On genus level, supplementation with ZnAA was able to prevent many of these losses (Figure 5C, right panel) (one way ANOVA followed by Tukey *post hoc* analysis). For example, no decrease in the Faecalibacterium genus that may be beneficial to the host concerning inflammatory processes (Sokol et al., 2008) was seen.

Interestingly, supplementation with ZnAA was able to prevent the loss of ZONULIN1 observed in mice on Zn Inhibitor diet (Figures 3C, 5D) (one way ANOVA, *p* = 0.0219; Tukey *post hoc* analysis: Control vs. Zinc Inhibitor, *p* = 0.0186; Control vs. Zinc inhibitor + ZnAA, *p* = 0.34). In addition, the levels of liver LPS decreased and were no longer significantly different from mice on control diet (Figure 5E) (one way ANOVA, *p* = 0.0065; Tukey *post hoc* analysis: Control vs. Zinc Inhibitor, *p* < 0.01; Control vs. Zinc inhibitor + ZnAA, *p* = 0.05738).

In the brain, we could not observe a significant effect on astrocyte activation, as animals on Zinc inhibitor + ZnAA diet still showed a significant increase in GFAP expression, although slightly less compared to mice on Zinc inhibitor diet (one way ANOVA, *p* = 0.0044; Tukey *post hoc* analysis: Control vs. Zinc Inhibitor, *p* = 0.0039; Control vs. Zinc inhibitor + ZnAA, *p* = 0.0219) (Figure 5F). In contrast, while mice on Zinc inhibitor diet had significantly increased IL-6 brain tissue levels, we detected no difference between Controls and mice on Zinc inhibitor diet supplemented with ZnAAs (Supplementary Figure S3B). Thus, zinc supplementation was able to prevent an increase in IL-6 brain levels.

## Discussion

Zinc deficiency plays a role in the etiology of depressive disorders in mouse models and humans. Several studies have reported an inverse relationship between low zinc levels and higher Hamilton Depression Rating Scale scores in patients (Maes et al., 1994). Interestingly, zinc deficiency also impairs the efficacy of several antidepressants (Tassabehji et al., 2008; Młyniec and Nowak, 2012; Młyniec et al., 2012). However, the mechanisms behind are not fully understood.

Further abnormalities have been independently reported in animal models and human patients with depression such as alterations in the gut microbiota composition and increased inflammatory responses and chronic inflammation. Several studies in the past revealed a link between depression and altered gut microbiota composition. From these a motif emerged, where significant alterations in the abundance of gut microbiota within the phyla *Bacteroidetes, Firmicutes, Proteobacteria*, and *Actinobacteria* were reported in patients diagnosed with major depressive disorder, but also in relevant rodent models (Winter et al., 2018).

With regards to inflammation, it was found that patients with major depressive disorder show all of the key features of an inflammatory response such as increased expression of pro-inflammatory cytokines and chemokines, cytokine receptors, and soluble adhesion molecules in peripheral blood and cerebrospinal fluid (CSF) (Miller and Raison, 2016). Especially, increased expression of IL-1β, IL-6, TNF, Toll-like receptor 3 (TLR3) and TLR4, has been found in post-mortem brains (Maes, 1995; Brambilla et al., 2014; Drago et al., 2015) and consistent with this, activation of IL-6, IL-8 and type I IFN-induced signaling pathways has been reported (Brambilla et al., 2014). A meta-analysis found that IL-1β, IL-6, TNF and C-reactive protein (CRP) in peripheral blood are the most reliable biomarkers of inflammation in patients with depression (Miller et al., 2009).

Here, we sought to establish a link between maternal zinc deficiency, altered microbiota composition, and inflammation. Zinc deficiency was reported before to affect microbiota composition. For example, similar to the result reported here, a decrease in Veruccomicrobia and an increase in Firmicutes has been observed (Mayneris-Perxachs et al., 2016). The low relative abundance of Verrucomicrobia populations and a decrease in beneficial bacteria was correlated with zinc deficiency in further studies (Lopez and Skaar, 2018). In addition, low zinc status as well as zinc supplementation were reported to affect gut microbiota in chicken (Reed et al., 2015, 2018). However, to our knowledge, the effects of zinc deficiency on the microbiome of pregnant mice in light of the observed behavioral alterations in the offspring of zinc-deficient mothers has not been investigated so far.

Using pyrosequencing of 16S rDNA of fecal samples, we obtained microbiota profiles from animals on four different diets: a control diet, a diet low in zinc, a diet with low bioavailability of zinc induced by elevated concentrations of other dietary components such as Fe, Ca, and folic acid that are commonly prescribed to pregnant women, and a diet with low bioavailability that was supplemented with zinc in the form of ZnAA to overcome inhibition by zinc uptake antagonists present in this diet (Sauer et al., 2017). Although no clear cut-off values between hypozincemia and zinc deficiency are established for mice, we consider the status of mice on a zinc-deficient diet and diet with zinc uptake inhibitors as mild zinc deficient. This is based on the fact that in many cases, in human studies, hypozincemia cannot be picked up in blood samples and blood/plasma zinc content is generally considered a poor measure of marginal zinc deficiency in humans (King, 1990; Wood, 2000). However, in our study, both animals on the zinc-deficient diet and diet with uptake inhibitors show significantly reduced zinc levels in blood. On the other hand, severe zinc deficiency was shown to induce gross anatomical malformation in pups from rats with severe zinc deficiency (Hurley et al., 1971). The pups born from pregnant mice in this study did have similar birth weight, no malformations and no statistically significant difference in the number of pups was detected in the different treatment groups compared to controls. Therefore, we conclude that the zinc deficiency we created was not severe but mild.

While both mice on a zinc-deficient diet and mice on a diet low in the bioavailability of zinc showed low tissue zinc levels and alterations in gut microbiota composition, the observed alterations in microbiota were not identical.

Mice on a zinc-deficient diet showed an increase in the phylum *Actinobacteria* and *Bacteroidetes*. *Actinobacteria* belong to the dominant commensal communities in humans and mice (Qin et al., 2010) and are generally regarded as pathobionts. Under certain circumstances, they are known to promote disease. In particular, *Actinobacteria* are associated with chronic inflammatory conditions and, for example, an increase in *Actinobacteria* has been associated with Inflammatory Bowel Disease (Frank et al., 2007; Morgan et al., 2012). However, mice on a diet with low bioavailability of zinc showed no such increase in *Actinobacteria* and *Bacteroidetes.*

Both groups of mice, however, showed an increase in *Firmicutes* and a decrease in *Proteobacteria.* In previous studies using mouse models for stress and depression-like behavior, an increase in *Actinobacteria* (Bangsgaard Bendtsen et al., 2012), both an increase and a decrease in *Bacteroidetes* (Aoki-Yoshida et al., 2016; Bharwani et al., 2016), an increase in *Firmicutes* (Aoki-Yoshida et al., 2016), as well as a decrease in *Proteobacteria* (Galley et al., 2014; Aoki-Yoshida et al., 2016) has been reported. Therefore, although different in some aspects both mice on a zinc-deficient diet and mice on a diet low in the bioavailability of zinc show alterations similar to those reported in models for stress and depression-like behavior.

The differences may originate in the competition between gut microbiota and enterocytic zinc uptake transporters for zinc. In mice on a zinc-deficient diet, a low amount of zinc is available for both. In contrast, in the diet with low bioavailability of zinc, zinc levels are normal but zinc uptake by enterocytic zinc transporters is inhibited through the antagonists present in the diet. Some microbiota may have an advantage over enterocytic zinc transporters with respect to inhibition by antagonists and may still be able to access sufficient amounts of zinc. For example, the phylum *Verrucomicrobia* was hardly affected by low bioavailability of zinc but reacted strongly to low general zinc levels. Further, due to low zinc levels, some families of bacteria may gain an advantage either by less demand for zinc, more sufficient intake mechanisms, or lack of competition through more zinc sensitive bacteria. For example, bacteria in the phylum *Firmicutes* were significantly increased in both zinc restricted diets. In addition, the presence of the zinc uptake inhibitors phytic acid, Ca, Fe, and folic acid may additionally influence microbiota. Therefore, it is not expected that a zinc-deficient diet and a diet with low bioavailability will alter microbiota composition in identical ways. However, besides shared and unique features in microbiota composition, both groups of animals show a reduction in tight junction markers and increased liver LPS levels. Thus, the shared aberrations from an established gut microbiota composition and/or low availability of zinc for GI cells are associated with pathological changes, such as increased permeability in the GI system in these mice.

Several studies support the idea that intestinal barrier dysbiosis leads to inflammatory responses in peripheral tissues and may ultimately drive inflammation in the brain. Therefore, we investigated the brain for characteristic alterations using GFAP and IL-6 as markers. We detected significantly higher GFAP expression in the brain of mice on zinc-restricted diets. Increased GFAP expression is a marker for activation on astrocytes and inflammation (Zhang et al., 2017). Our results are in line with previous reports that acute stress increases GFAP expression in the hippocampus of rodents (Lambert et al., 2000). Zinc deficiency may physiologically act as an acute stressor.

IL-6 plays a key role in the development of stress-associated depression-like behaviors in mice (Chourbaji et al., 2006). IL-6 signaling can result from activation of inflammatory pathways and alterations in IL-6 levels in the brain were demonstrated contributing to depression symptomatology (Hodes et al., 2016). Indeed, IL-6 is consistently reported as elevated in the blood of patients with depression (Haapakoski et al., 2015) and has been proposed as a predictive biomarker. Therefore, here, we assessed IL-6 levels in the brain mice. Our data show increased IL-6 tissue levels on mRNA and protein levels in response to low zinc status in pregnant mice. These results are in line with previously reported results showing up-regulation of cell activation markers in THP1 cells, a model for human monocytes, that coincided with increased IL-6 responses following LPS stimulation (Wong et al., 2015). In addition, a decreased zinc status in aged mice was associated with increased IL-6 expression levels (Wong et al., 2015).

Finally, supplementation of the diet with low bioavailability of zinc with ZnAAs was investigated to validate the contribution of zinc deficiency to the observed alterations and to understand the usability of ZnAAs for zinc supplementation during pregnancy. Supplementation with ZnAAs was able to prevent a significant drop in zinc levels in the maternal blood. In terms of microbiota composition, the presence of the ZnAA supplement is not expected to create a similar condition as observed in controls due to the presence of zinc uptake antagonists and increased zinc levels. However, supplementation with ZnAA may reverse some effects caused by lowered bioavailability of zinc due to the presence of the antagonists. Indeed, ZnAA supplementation ameliorated the decrease in *Proteobacteria.* In addition, supplementation with ZnAAs prevented the decrease in *Actinobacteria* and *Bacteroidetes*, which was specific for this diet. However, it leads to an increase of both phyla as observed in the zinc-deficient diet before. Both phyla, therefore, seem to respond very sensitive to zinc levels and possibly contain species that thrive in low zinc conditions and others that strive with high zinc levels. Therefore, it is more important to investigate alterations on the genus and species level. Here, ZnAA supplementation ameliorated alterations observed before in several genera such as *Anaertruncus, Acetifactor, Desulfonispora, Holdemanella, Rhodospirillum*, and *Desulfovibrio*.

In terms of GI pathology, we could no longer detect an increase in ZONULIN1 levels in ZnAA supplemented mice and, in line with this, no significant increase in liver LPS. Thus, effects on gut physiology seem indeed to be dependent on zinc availability much more than on microbiota composition and it can be assumed that alterations in microbiota composition are a consequence of altered GI function or dependent on dietary factors only, or both. The reduction in GI abnormalities and liver LPS are expected to decrease pro-inflammatory processes in the mice. While we could not detect a normalization of GFAP expression, IL-6 protein levels in brain tissue were indeed normalized. While IL-6 levels in the brain of humans are difficult to measure, a reduction of IL-6 levels in plasma after zinc supplementation has been reported also in humans before (Bao et al., 2010). The data further confirms that ZnAAs are not only increasing zinc levels in animals but that they are biologically active.

Taken together, we conclude that both low levels of zinc or the presence of zinc uptake inhibitors that are commonly found in western diets and supplements for pregnant women alter the microbiome of pregnant mice. This may not only play a role in the observed autism-like phenotype of the offspring of mice with zinc deficiency during pregnancy but may also directly influence brain functionality through altered gut-brain signaling. Low zinc status was associated with changes in the intestinal epithelial barrier and an increase in liver LPS hints at increased leakiness of the gut in response to these changes. Finally, this may contribute to increased inflammation as we have observed higher GFAP and IL-6 levels in the brain of mice. Acute zinc deficiency was linked to depression and a role of microbiota dysbiosis and inflammation suggested. Our results obtained from pregnant mice do not exclude that similar alterations may occur independent of pregnancy in response to low zinc status. Based on our results, as low availability of zinc during pregnancy influences both microbiota and inflammatory status, a link between maternal zinc deficiency and postpartum depression seems plausible.

## Data Availability Statement

Additional datasets generated for this study are included in the Supplementary Material, and all datasets are available on request.

## Ethics Statement

All animal experiments were performed in accordance with the guidelines and regulations for the welfare of experimental animals issued by the Federal Government of Germany and by the local ethics committee (Ulm University). The protocol used was approved by the Regierungspräsidium Tübingen, state of Baden-Württemberg, and the Ethics Committee of Ulm University (ID Number: 1257).

## Author Contributions

AS carried out the analysis of mice and revised the manuscript. AG conceived the study, participated in its design, coordination, and data analysis, and drafted the manuscript. All authors read and approved the final manuscript.

## Conflict of Interest

The authors declare that the research was conducted in the absence of any commercial or financial relationships that could be construed as a potential conflict of interest.
