# Supplementary material for: Zinc Deficiency During Pregnancy Leads to Altered Microbiome and Elevated Inflammatory Markers in Mice
Source: Front Neurosci. 2019 Nov 29;13:1295. doi: 10.3389/fnins.2019.01295 (PMC6895961; doi:10.3389/fnins.2019.01295)
Supplement: Supplementary file 1 [file Data_Sheet_1.PDF]

Zinc deficiency during pregnancy leads to altered microbiome and elevated inflammatory markers in mice

Ann Katrin Sauer, Andreas M. Grabrucker

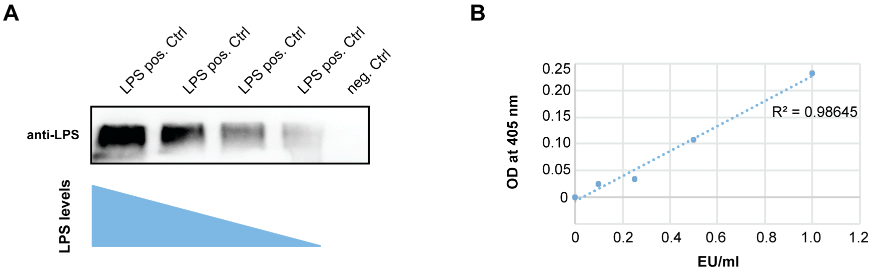

Figure S1: **A)** Lysate containing LPS (pos. Ctrl, lane 1-4) and lysis buffer without addition of LPS (neg. Ctrl, lane 5) were used for western blotting. Using the anti-LPS antibody, LPS was detected with bands corresponding to the LPS concentration used. The lane with the sample without LPS (neg. Ctrl) showed no signal. **B)** Using an *E.coli* LPS, Standard Stock Solutions were prepared yielding 0 endotoxin units / ml (EU/ml), 0.1 EU/ml, 0.25 EU/ml, 0.5 EU/ml, and 1.0 EU/ml. The blank contained 50 µl of endotoxin-free water. LAL and Chromogenic Substrate solution were added according to the manufacturer's protocol and absorbance measured at 405 nm on a plate reader. A standard curve was plotted using the average blank-corrected absorbance for each standard on the y-axis. Using the formulated standard curve (linear regression), LPS concentrations were determined in liver tissue lysate of mice.

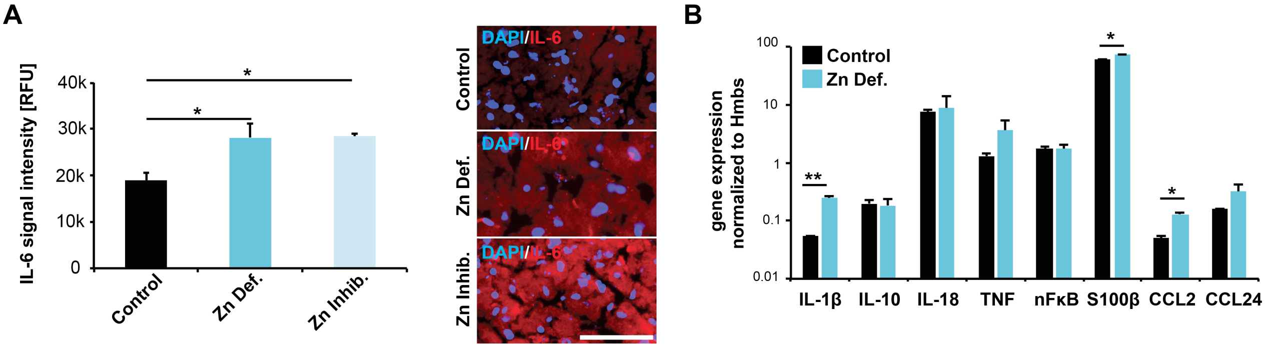

Figure S2: **A)** Brain sections from three animals per group were used for immunohistochemistry. DAPI (labeling cell nuclei) and IL-6 was visualized and fluorescent signal intensities from 10 cells in the hippocampus from three sections per animal measured. Mean values show the average of three animals per group. A significant increase in IL-6 tissue levels can be detected in the brains of mice subjected to zinc deficiency and lowered bioavailability (Zn Inhibitor) compared to control mice. Scale bar = 100 µm. **B)** Whole-brain mRNA was obtained from 3 mice per group and qRT-PCR analyses performed in triplicates. Gene expression is shown normalized to Hmbs. Marker genes of the interleukin family (IL-1b, IL-10, IL18), chemokine ligands (CCL2, CCL24), TNF, nFκB, and the damage associated pattern (DAMP) S100β were measured. A significant increase in IL-1b (p = 0.0034), S100β (p = 0.0185), and CCL2 (p = 0.0109) mRNA expression levels was detected in mice on a zinc-deficient diet (t-tests).

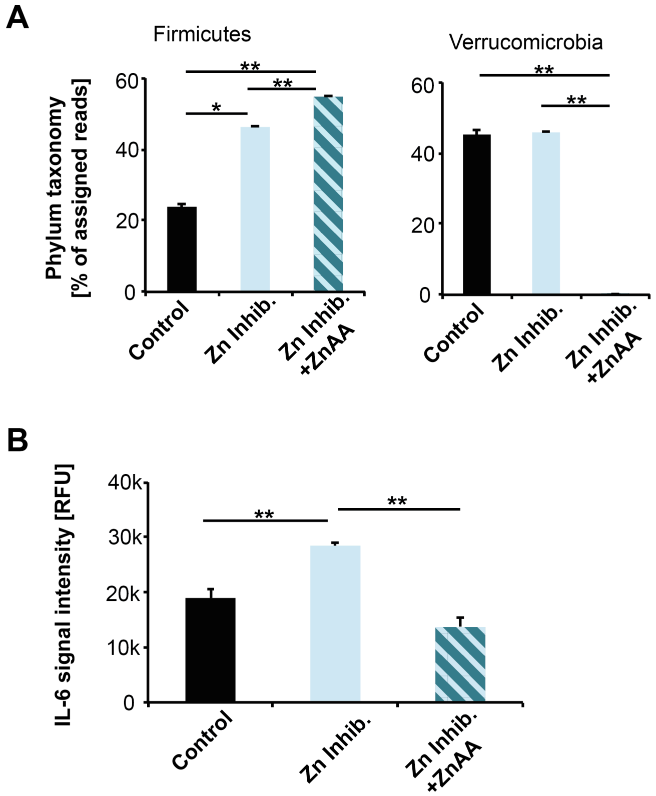

Figure S3: A) DNA was extracted from feces and microbiota composition analyzed using 16s microbiome profiling. Using the obtained sequence information, single species and their relative amounts were identified. Mice on both Zn inhibitor diet and Zn Inhibitor + ZnAAs diet had significantly higher levels of Firmicutes. ZnAA supplementation significantly reduced levels of Verrucomicrobia. Actinobacteria were significantly lower in mice on Zn inhibitor diet compared to controls and mice on Zn inhibitor diet + ZnAA. The levels were significantly higher in mice on Zn Inhibitor + ZnAAs diet compared to controls. Levels of Bacterioidetes were significantly increased in mice on Zn inhibitor diet + ZnAA compared to the control and Zn inhibitor diet. **B)** Brain sections from three animals per group were used for immunohistochemistry. DAPI (labeling cell nuclei) and IL-6 was visualized and fluorescent signal intensities from 10 cells in the hippocampus from three sections per animal measured. Mean values show the average of three animals per group. The significant increase in IL-6 levels under low bioavailability of zinc was reversed upon supplementation with ZnAAs. A significant difference was detected between IL-6 tissue levels of mice on Zn inhibitor diet and mice and Zn inhibitor diet + ZnAAs (one way ANOVA, $p = 0.0009$; Tukey post-hoc analysis: Control vs. Zinc Inhibitor, $p = 0.0075$; Zinc Inhibitor vs. Zinc inhibitor + ZnAA, $p = 0.001005$).
